# A Gas Sensor With Fe_2_O_3_ Nanospheres Based on Trimethylamine Detection for the Rapid Assessment of Spoilage Degree in Fish

**DOI:** 10.3389/fbioe.2020.567584

**Published:** 2020-09-23

**Authors:** Likun Liu, Shuang Fu, Xiao Lv, Liling Yue, Li Fan, Haitao Yu, Xiuli Gao, Wenbin Zhu, Wei Zhang, Xin Li, Wenquan Zhu

**Affiliations:** ^1^Research Institute of Medicine and Pharmacy, Qiqihar Medical University, Qiqihar, China; ^2^College of Pharmacy, Qiqihar Medical University, Qiqihar, China; ^3^Procurement Management Office, General Hospital of Heilongjiang Province Land Reclamation Bureau, Harbin, China; ^4^The Third Affiliated Hospital, Qiqihar Medical University, Qiqihar, China

**Keywords:** solvothermal method, nanoparticle, α-Fe_2_O_3_, trimethylamine gas sensing, fish spoilage

## Abstract

A spherical iron oxide precursor was prepared using a solvothermal method, and then treated thermally at 400°C to obtain α-Fe_2_O_3_ nanoparticles. The structures and morphology of the as-obtained products were characterized using X-ray diffraction (XRD), transmission electron microscopy (TEM), and scanning electron microscopy (SEM). The results showed that the diameter of the α-Fe_2_O_3_ nanoparticles was approximately 500 nm. In addition, we formed the α-Fe_2_O_3_ nanoparticles into a thick film as a gas sensor and performed a gas sensing test. When the working temperature was set at 250°C, the α-Fe_2_O_3_ nanoparticle displayed very good selectivity and high sensitivity for trimethylamine (TMA). The minimum detection was as low as 1 ppm, and the response value for 100 ppm TMA gas was 27.8. Taken together, our findings illustrated that the α-Fe_2_O_3_ nanoparticles could be used as a gas-sensitive material to test the freshness of fish.

## Introduction

Fish is considered as an excellent human food because it not only provides high quality protein and a wide variety of vitamins and minerals (Efsa Dietetic Products Nutrition and Allergies, 2014), but also is the main dietary source of Omega-3 polyunsaturated fatty acid (ω-3 PUFAs) which are well known for their anti-oxidant and anti-inflammatory effects (Chen et al., 2017; Vilavert et al., 2017). However, fresh fish is highly perishable because of enzymatic autolysis, lipid oxidation and rapid microbial growth which are present in the fish or are acquired from environmental contamination (Ghaly et al., 2010; Jiang et al., 2018). Among them, microbial growth is thought to be the primary reason for the deterioration of fish quality (Hassoun and Çoban, 2017). Trimethylamine oxide (TMAO), which is naturally present in almost all marine fish and some freshwater fish can be rapidly converted rapidly into trimethylamine (TMA) by the action of bacteria during spoliage (Chaillou et al., 2015; Klouptsi et al., 2018). TMA, as a volatile base, is largely responsible for the characteristic of fish spoilage. It was confirmed that TMA levels in tissue gradually increased as fish freshness decreased (Mitsubayashi et al., 2004; Rappert and Muller, 2005). Therefore, the TMA level can be used as a key indicator for fish freshness (Sun et al., 2019).

Several techniques have be developed to detect TMA including sensory testing (Egashira et al., 2002; Li and Lee, 2007), chemical testing (Guo et al., 2013), *K* value determination (Ehira and Uchiyama, 1987) and biosensor methods (Ghosh et al., 1998). However, all these methods suffer from drawbacks, such as the need for the operators to have long-term experience, a requirement for expensive auxiliary analytical instruments, complicated operation, short service life and poor stability. For these reasons, a sensitive, cheap, accurate and fast method to detect TMA is required.

The assessment of fish freshness using a gas sensor based on a metal oxide semiconductor material that detects TMA represents a non-destructive and rapid detection method, and has been studied widely. This method has the advantages of high sensitivity, low cost, convenient and rapid detection process, and has played an important role in rapid and non-destructive detection of fish freshness (Chen et al., 2014; Chang et al., 2017; Wang et al., 2018; Zhang et al., 2019). Nano-α-Fe_2_O_3_ is a typical n-type semiconducting metal oxide with a forbidden band width of 2.2 eV. It is inexpensive and causes no environmental pollution (Zeng et al., 2015; Wang and Huang, 2016). Although α-Fe_2_O_3_ nanosensor materials with different morphologies have been used to detect gasses such as H_2_S (Li et al., 2015; Teng et al., 2020), acetone (Jin et al., 2015), ethanol (Wang et al., 2015), triethylamine (Song et al., 2018), there are few reports (Hu et al., 2016) on the actual detection of the freshness of fish-based products. The development of new α-Fe_2_O_3_ nanomaterials based on the detection of TMA for rapid, non-destructive and accurate detection of fish freshness is of great significance for preventing food spoilage and ensuring food safety. In this study, pure phase α-Fe_2_O_3_ nanospheres were prepared using a simple solvothermal synthesis method, and their structures and morphologies were characterized using X-ray diffraction (XRD), transmission electron microscopy (TEM), and scanning electron microscopy (SEM). The α-Fe_2_O_3_ nanometers were investigated and the gas-sensitive properties of the microspheres to TMA were tested to assess the freshness of freshwater fish.

## Materials and Methods

### Synthesis of the α-Fe_2_O_3_ Nanoparticles

Iron acetylacetonate (0.12 g) and glucose (0.0065 g) were dissolved in 30 mL of *n*-butanol and stirred for 10 min. The mixture was subjected to ultrasonication to obtain a dark red transparent solution. Subsequently, 5 mL 1 M of nitric acid solution was added into the mixture and stirring was continued for 1 h. The obtained solution was poured into a 1–50 mL volumetric stainless steel reactor with a Teflon liner and heated at 180°C for 12 h. Thereafter, the reactor was cooled naturally to room temperature. The resultant red precipitate was collected and washed three times with deionized water and ethanol respectively before being dried in an oven at 70°C for 12 h. Finally, a pure α-Fe_2_O_3_ powder named α-Fe_2_O_3_-pre was obtained after calcination in a muffle furnace at 400°C for 2 h.

### Characterization

SEM (Hitachi S-4300; Hitachi, Tokyo, Japan) and TEM (EM-208S; CSIS, United States) were adopted to evaluate the morphology and size of α-Fe_2_O_3_ nanoparticles. The pore size distribution and adsorption-desorption curves of the α-Fe_2_O_3_ nanoparticles were measured using a nitrogen physical adsorption instrument (VSorb 2800P; Gold APP Instruments, China). Their crystalline structure and phase composition were analyzed using an X-ray diffractometer (PW3040/60; Philips, the Netherlands). To perform SEM analysis, the α-Fe_2_O_3_ nanoparticles were sprayed in a vacuum environment to coat the metal strip and then placed under the scanning electron microscope for observation. To perform TEM analysis, a small amount of nanoparticles was fixed on the copper grid for characterization. To perform nitrogen adsorption-desorption analysis, α-Fe_2_O_3_ nanoparticles were placed in liquid nitrogen after removed the physically adsorbed moisture at 55°C. The pore size distribution and adsorption-desorption curves were measured using the Nitrogen physical adsorption instrument. To perform XRD analysis, a small amount of samples was placed in the ray scanning bath. The scan rate was 5°/min. Scan angles ranged from 20° to 80°.

### Preparation of the α-Fe_2_O_3_ Sensor

An appropriate amount of α-Fe_2_O_3_ nanoparticles was mixed with several drops of the monoterpene alcohol terponepl until a paste was formed. The paste was then coated onto the surface of an alumina ceramic tube with a gold electrode, dried in an oven at 70°C for 30 min and then calcinated in a muffle furnace at temperatures from 300 to 600°C for 1 h. Finally, a Ni-Cr alloy coil heating wire was inserted into the alumina ceramic tube to form a gas sensor. The thickness of the sensing films was about 100 μm. The alumina ceramic tube was then welded to a pedestal, and the obtained sensors were aged at 250°C for 7 days to form the final sensor unit. The sensors obtained were named as α-Fe_2_O_3_-300, α-Fe_2_O_3_-400, α-Fe_2_O_3_-500, and α-Fe_2_O_3_-600, according their calcination temperatures, respectively.

### Gas-Sensing Performance Test

A gas sensing test of the sensors was carried out using an improved static gas distribution method. When the resistance of sensors was stable, a certain amount of TMA was injected into a 10 L vacuum glass vessel using a syringe. After measurement, the sensor was exposed to air by opening the glass vessel to the atmosphere. The gas sensitivity characteristic of α-Fe_2_O_3_ sensor was tested using a JF02E (Kunming, China) gas sensor characteristic tester. The relative humidity of the test was 35 ± 5 RH%, the temperature was 24 ± 1°C, and the sensor response value to TMA was calculated as S = Ra./Rg, where Ra and Rg are the resistance values of the gas sensor when it was stabilized in the air and in the gas to be measured at the working temperature, respectively.

### Effect of Operating Temperature on the Gas Sensing Properties of the Sensor

To investigate the effect of calcination temperature on the gas sensing properties, the α-Fe_2_O_3_ sensors obtained at different calcination temperatures (300, 400, 500, and 600°C) were placed in 100 ppm of TMA gas, respectively. The measurement was carried out using the method detailed in “Gas-Sensing performance test.”

### Effect of Gas Type on Gas Sensing Properties of Sensor

To further investigate the effect of gas type on gas sensing properties, the α-Fe_2_O_3_ sensor was placed in different gases including carbon monoxide, ammonia, H_2_S, TMA, ethanol, and acetone for gas sensitivity testing. The gas concentration was set to 100 ppm, and the working temperature was 250°C. The measurement was carried out using the method detailed in “Gas-Sensing performance test.”

### Effect of TMA Concentration on the Gas Sensing Properties of the Sensor

To investigate the effect of TMA concentration on the gas sensing properties, the α-Fe_2_O_3_ sensor was placed in different concentrations of TMA gas to determine its gas sensitivity. The measurement was carried out using the method detailed in “Gas-Sensing performance test.”

### The Response and Recovery Time Study

The response and recovery curves of the nano-microsphere α-Fe_2_O_3_ thick film elements were measured using the resistance response method. The response time was the time taken when the resistance value was decreased by Ra-90% (Ra-Rg) from the initial Ra after the gas sensor contacted the gas to be measured. The recovery time was the time taken for the resistance value to increase from Rg to Rg + 90% (Ra-Rg) after the gas sensor was separated from the gas to be measured.

### Effect of Relative Humidity on the Gas Sensing Properties of the Sensor

The gas sensing properties of the α-Fe_2_O_3_ sensor were tested under different humidity conditions (RH = 30, 55, 75, and 95%). The working temperature was set as 24 ± 1°C. The measurement was carried out using the method detailed in “Gas-Sensing performance test.”

### Stability of the Sensors

To investigate the stability of the α-Fe_2_O_3_ sensor, gas sensing at different TMA concentrations (10 and 100 ppm) were performed for 60 days, at a test frequency once every 10 days.

### Fish Freshness Detection

Ten live fresh freshwater carp were bought from a local market, each of which was about 150 g. One fish was killed quickly by a sharp collision of the brain, and the rest of the fish was not treated. The fish were placed in an air-conditioned environment at 25°C, then transferred to a glass container and sealed for gas sensitivity testing. The concentration of TMA was measured when the sensor was placed in the glass container. The sealing plug was then opened to allow the fish to be in the same atmosphere as the sensor. The TMA concentration was tested every 4 h in the same way to examine the relationship between fish freshness and the release of TMA.

## Results and Discussion

### Morphology and Microstructure of α-Fe_2_O_3_-pre

**FIGURE 1:**
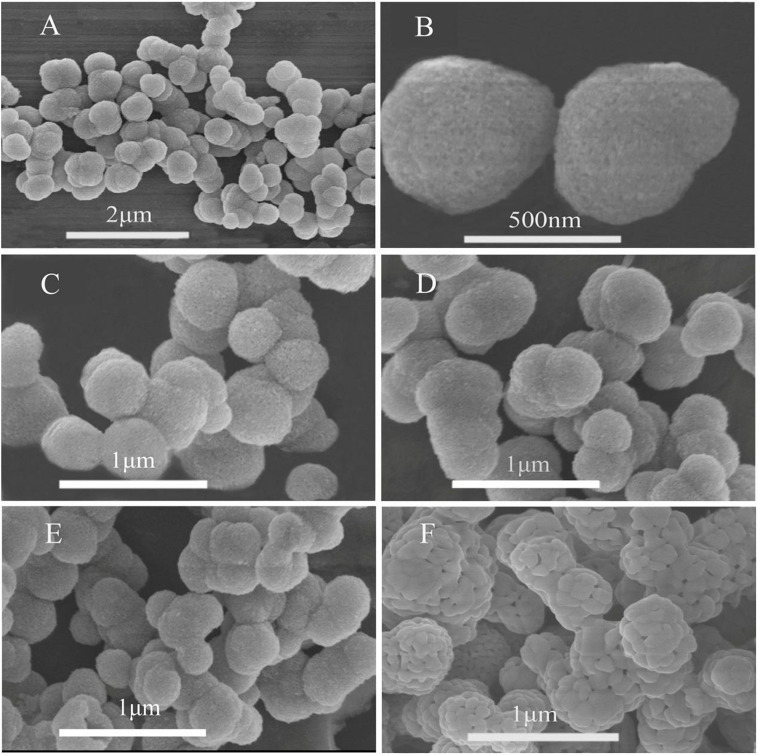
Scanning electron microscopy (SEM) micrograph of **(A)** α-Fe_2_O_3_-pre, **(B)** α-Fe_2_O_3_-pre (amplification), **(C)** α-Fe_2_O_3_-300, **(D)** α-Fe_2_O_3_-400, **(E)** α-Fe_2_O_3_-500, and **(F)** α-Fe_2_O_3_-600.

The morphology of the α-Fe_2_O_3_ nanoparticles was characterized using SEM. As shown in Figure 1A, the α-Fe_2_O_3_-pre had a spherical shape. The average particle diameter was uniform at approximately 500 nm. The SEM image at a higher magnification (Figure 1B) showed that the surface of the α-Fe_2_O_3_-pre is uniformly deposited with small particles, and the particles are filled with tiny holes, which may be beneficial for the gas-sensitive properties of the α-Fe_2_O_3_ sensor. Figures 1C–E shows that there was no agglomeration and melting change of the precursor when calcinated at 300, 400, and 500°C, while the α-Fe_2_O_3_-pre in Figure 1F melted after being calcinated at 600°C for 2 h and their surface pores were also blocked. The above results showed that the calcination temperature had a significant effect on the pore structure of α-Fe_2_O_3_ which is important for its gas-sensitive properties.

**FIGURE 2:**
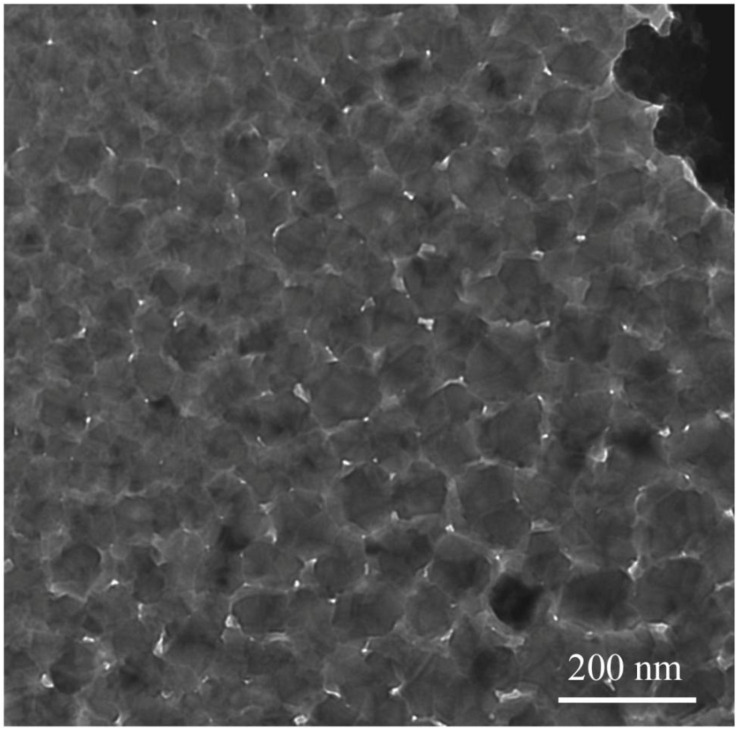
Transmission electron microscopy image of synthesized α-Fe_2_O_3_ nanoparticles.

Transmission electron microscopy was employed to gain further insights into the morphological features of α-Fe_2_O_3_-pre. Figure 2 presents a TEM image of α-Fe_2_O_3_-pre. The synthesized α-Fe_2_O_3_-pre comprises many nanoparticles with regular shapes. The average size is about 500 nm, which is in agreement with the value indicated by SEM analysis.

**FIGURE 3:**
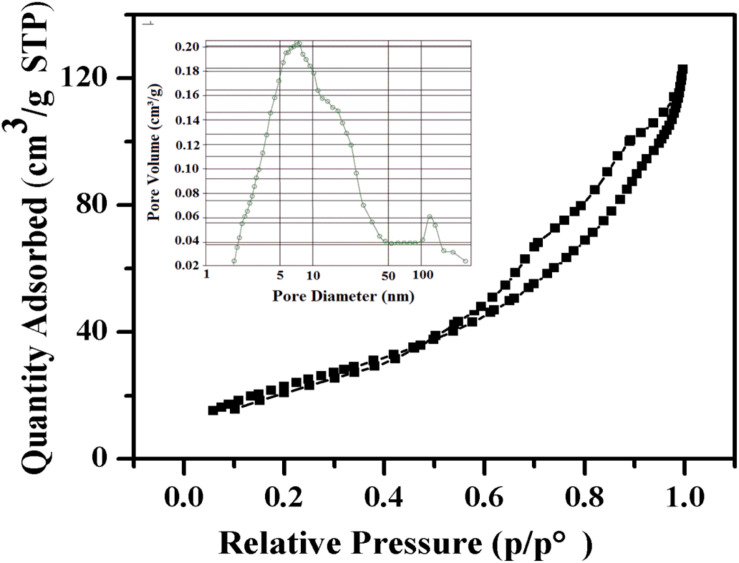
Nitrogen adsorption-desorption isotherm and BJH pore size distribution (inset) of the α-Fe_2_O_3_ nanoparticles.

Nitrogen adsorption analysis was used to determine the surface voids of α-Fe_2_O_3_-pre. As shown in Figure 3, the N_2_ adsorption-desorption isotherm curves of α-Fe_2_O_3_-pre show an obvious receipt ring, indicating that it has a mesoporous structure. In addition, the average pore size of the α-Fe_2_O_3_ nanospheres was about 7.71 nm, and their pore size distribution is concentrated. Moreover, the BET surface area of the α-Fe_2_O_3_ nanospheres is 88.86 m^2^/g. A large specific surface area is also an important factor for the gas sensing properties of the α-Fe_2_O_3_ sensor.

**FIGURE 4:**
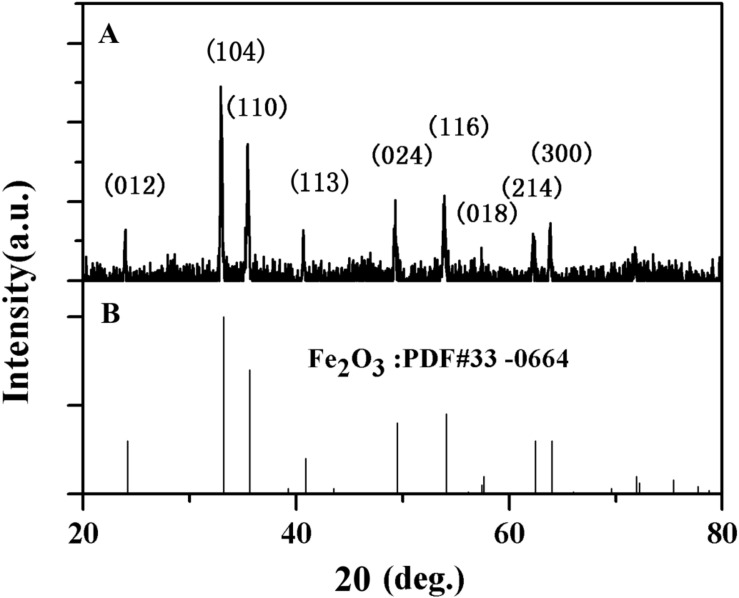
X-ray diffraction (XRD) pattern of α-Fe_2_O_3_ nanoparticles **(A)** Synthesized and **(B)** Purified (JCPDS No.33 -0664 Standard card).

The structure of α-Fe_2_O_3_-pre was characterized by XRD, as shown in Figure 4, the crystal diffraction peaks of α-Fe_2_O_3_-pre are completely identical with those of the standard card, and no other additional diffraction peaks were observed. This suggested that there are no significant impurities.

### Sensing Properties of the α-Fe_2_O_3_ Sensor

**FIGURE 5:**
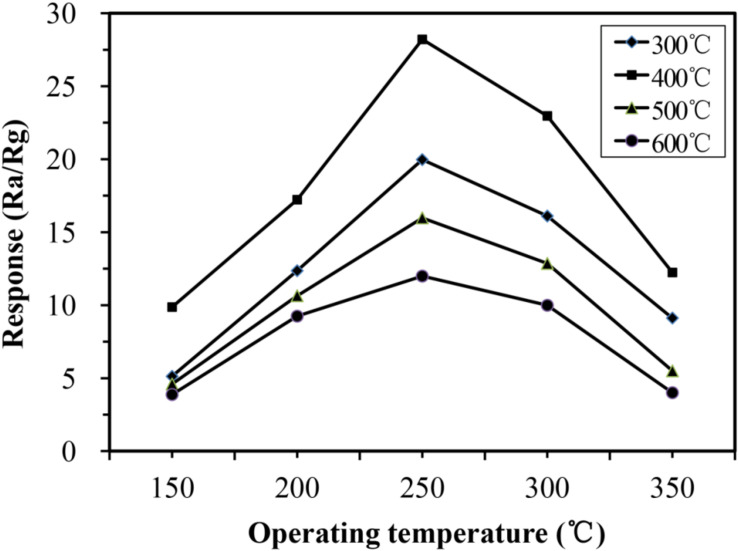
Responses of α-Fe_2_O_3_-300, α-Fe_2_O_3_-400, α-Fe_2_O_3_-500, and α-Fe_2_O_3_-600 sensor to 100 ppm trimethylamine (TMA) at different temperatures.

The operating temperature is an important factor that affects the gas-sensing performance of gas sensors. To find the optimum temperature for TMA detection, the temperature-dependent responses to 100 ppm TMA gas were investigated from 150 to 350°C. As shown in Figure 5, the responses of all samples increased with increasing operating temperature, reaching the maximum at 250°C, and then decreasing as the temperature continued to increase from 250 to 350°C. This suggested that the operating temperature should be set at around 250°C. This phenomenon could be explained as follows: When the temperature is too low, the response toward TMA is small because of insufficient activation of the nano-rods; however, too high temperatures lead to the escape of some adsorbed TMA molecules before their reaction with Fe_2_O_3_, resulting in decreased responses (Sakai et al., 2001). The sensitivity to 100 ppm TMA of the α-Fe_2_O_3_ sensor calcinated at 400°C was significantly higher than that of the sensor calcinated at 300, 500, or 600°C. This result confirmed that calcination at 400°C is more conducive to the detection of TMA by α-Fe_2_O_3_ nanospheres.

**FIGURE 6:**
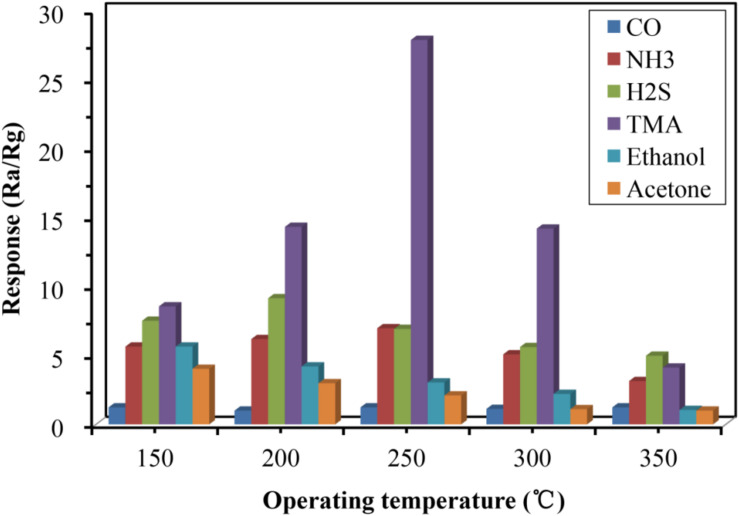
Responses of the developed α-Fe_2_O_3_ sensor to a variety of substances including TMA, CO, NH_3_, H_2_S, ethanol, and acetone.

Studies have shown that α-Fe_2_O_3_ sensors could be used to detect a variety of gases, including CO, NH_3_, and H_2_S, and several volatile organic compounds, such as ethanol and acetone. Therefore, it was essential to test the selectivity of the developed α-Fe_2_O_3_ between TMA and these other substances. As shown in Figure 6, the α-Fe_2_O_3_ sensor responded to CO, NH_3_, H_2_S, ethanol, and acetone with a response value of less than 10, while the response value to TMA reached 27.8. We conclude that the α-Fe_2_O_3_ sensor has very good selectivity and high sensitivity to TMA at a concentration of 100 ppm at 250°C. We speculated that the high affinity of TMA for Fe_2_O_3_ contributes to the selectivity and high sensitivity of the α-Fe_2_O_3_ sensor.

**FIGURE 7:**
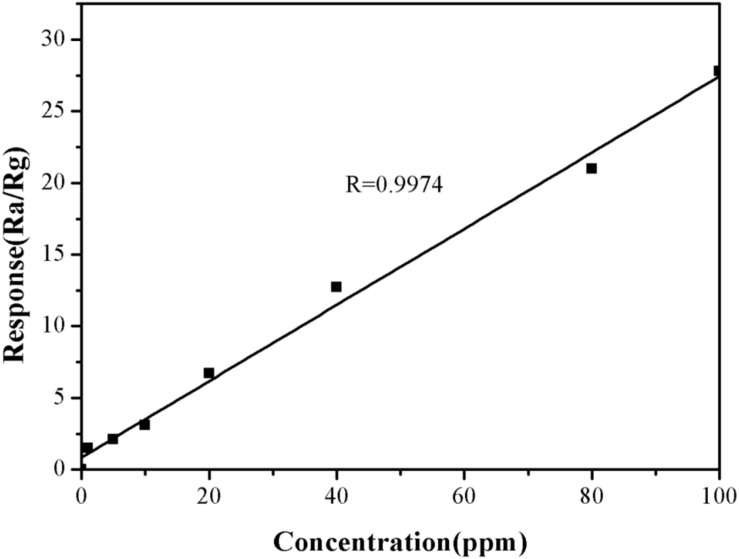
Response and recovery times of the α-Fe_2_O_3_ sensor to TMA gas (1–100 ppm) at the operating temperature of 250°C.

**FIGURE 8:**
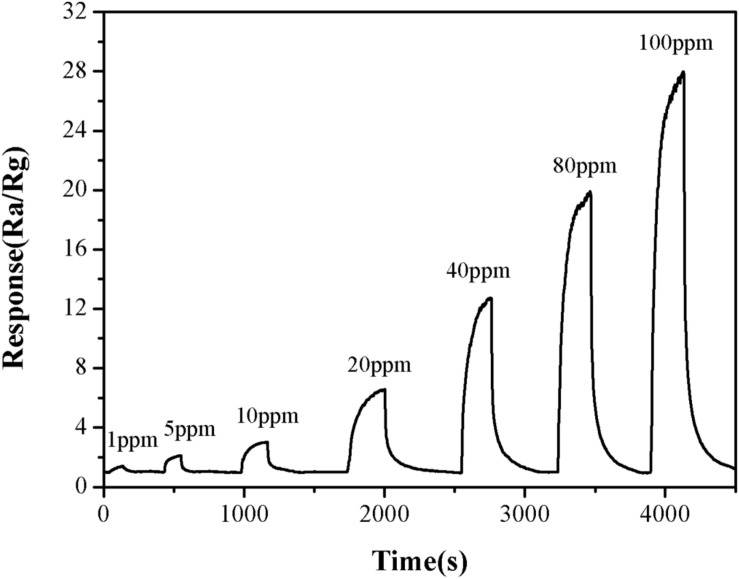
Responses of the developed α-Fe_2_O_3_ sensor to different concentrations of TMA (1–100 ppm) at the operating temperature of 250°C.

The effect of TMA concentration on the sensitivity of the α-Fe_2_O_3_ sensor was also investigated. The response value for TMA gas at 1 ppm was 1.5. When the concentration of TMA gas was increased to 100 ppm, the response value for TMA gas was 27.8 (Figure 7). This suggested that the higher the TMA concentration, the greater the response value of the nanomicrosphere α-Fe_2_O_3_ would become. In addition, in the concentration range of 1–100 ppm, there was a good linear relationship (*R* = 0.9974) between the response value of the α-Fe_2_O_3_ sensor to TMA and its concentration (Figure 8). Therefore, the developed α-Fe_2_O_3_ sensor could be used to detect TMA in complex environments.

The response and recovery time of the α-Fe_2_O_3_ sensor would directly determine its detection efficiency. Therefore, the response and recovery curves of the α-Fe_2_O_3_ sensor were measured using the resistance response method. The results (Figure 7) showed that the α-Fe_2_O_3_ nanospheres responded to TMA gas within 1 min, and its recovery time was less than 4 min. Thus, the response and recovery time of the sensor could meet the needs of practical applications.

**FIGURE 9:**
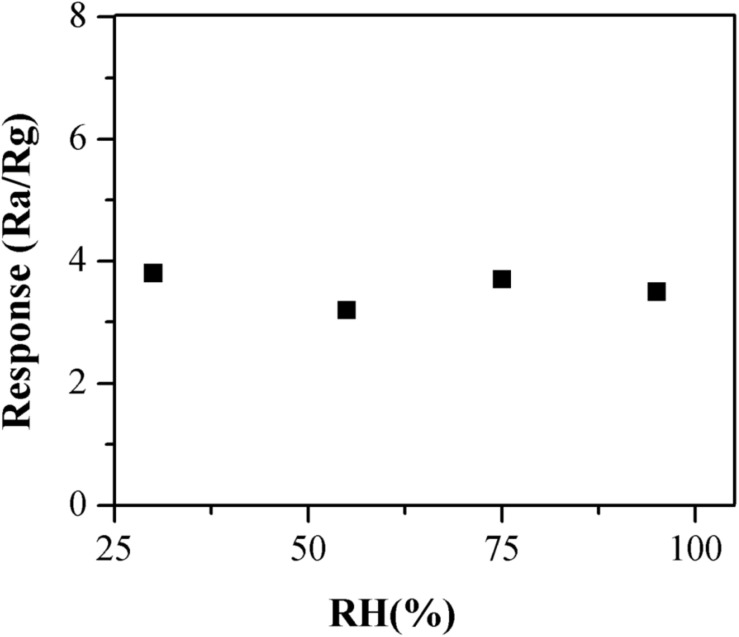
The moisture resistance of the α-Fe_2_O_3_ sensor under different humidity conditions (RH = 30, 55, 75, and 95%).

The practical application of the α-Fe_2_O_3_ sensor to detect TMA gas generally occurred in a humid environment such as a fishery market or a supermarket. The humidity is usually higher because of the operating environment. Therefore, the α-Fe_2_O_3_ sensor’s moisture resistance could have a significant impact on its detection sensitivity. The gas sensitivity of the α-Fe_2_O_3_ sensor was tested under different humidity conditions (RH = 30, 55, 75, and 95%). As shown in the Figure 9, the results obtained under different humidity conditions were not significantly different, indicating that the sensor was insensitive to humidity and was resistant to moisture. This might have been caused by the high working temperature such that the water molecules adsorbed by the sensor evaporated and air humidity surrounding the sensor was also reduced.

**FIGURE 10:**
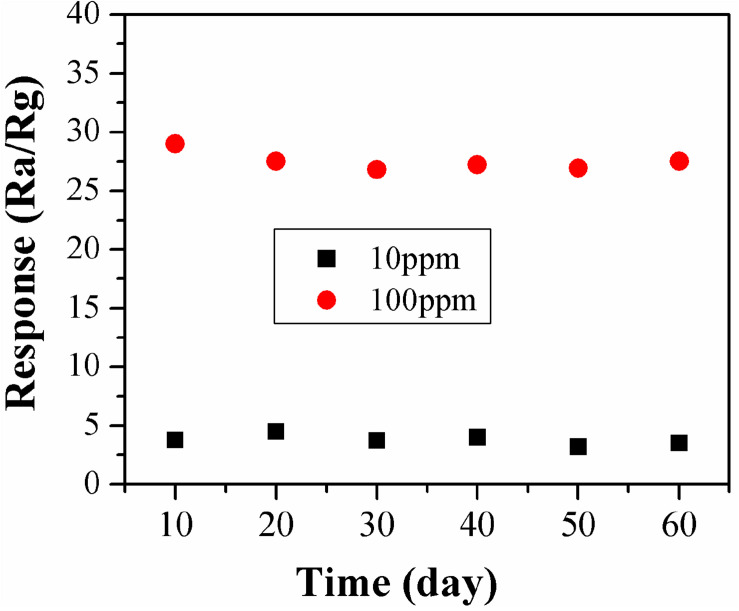
Responses of α-Fe_2_O_3_ sensor to TMA gas (10 and 100 ppm) over 60 days.

Long-term stability of a gas sensor is very important for its practical application. Therefore, the stability of the α-Fe_2_O_3_ sensor was investigated. The results are showed in Figure 10. The response of the sensor to TMA gas was almost constant over 60 days, indicating that the α-Fe_2_O_3_ sensor could measure the TMA gas stably.

### Gas-Sensing Mechanism

The TMA sensing mechanism on α-Fe_2_O_3_ nanoparticles based sensor was described as follows: α-Fe_2_O_3_ was a typical n-type metal oxide semiconductor. For n-type semiconductor sensor, its sensing performance was controlled by the variation of the electrical resistance that resulted from the interaction between TMA and the oxygen species absorbed on the surface of sensor (Kim and Yong, 2011; Li et al., 2015). That means the sensing performance of the α-Fe_2_O_3_ sensor is highly correlated with its adsorbing oxygen ability. When the α-Fe_2_O_3_ sensor was exposed to the air, the oxygen molecules were adsorbed on the surface of the α-Fe_2_O_3_ and became adsorbed oxygen.

The adsorbed oxygen trapped free electrons from the conductance band of the semiconductor at elevated temperature and formed negatively charged chemisorbed oxygen species (O_2_^–^, O^–^, O^2–^). This might lead to a decrease in the conductivity of the sensor and an increase of energy band, and then an electron-depletion layer was formed in the surface, further generated a barrier. The resistance of the α-Fe_2_O_3_ sensor was increased. It is noteworthy that the chemisorbed oxygen species highly depend on the working temperature. When the temperature is below 100°C, O_2_^–^ is usually chemisorbed. At higher temperatures (over 100°C), O^–^ and O^2–^ become commonly chemisorbed while O_2_^–^ disappears quickly (Chang et al., 2008). When the TMA was introduced to the test chamber, TMA could react with adsorbed oxygen species (O_2_^–^, O^–^, O^2–^) on the surface of the α-Fe_2_O_3_ sensor, and the captured electrons would return to the conduction band. As a result, the conductivity of the sensor increased, the electron-depletion layer and the barrier were reduced, finally caused a decrease in the surface resistance of the α-Fe_2_O_3_ sensor. In the present study, the α-Fe_2_O_3_ sensor displayed a fast response toward TMA mainly due to its porous structure which was benefit for the diffusion of TMA to the surface of α-Fe_2_O_3_ sensor.

### Application of α-Fe_2_O_3_ Sensor in Detecting Fish Freshness

**FIGURE 11:**
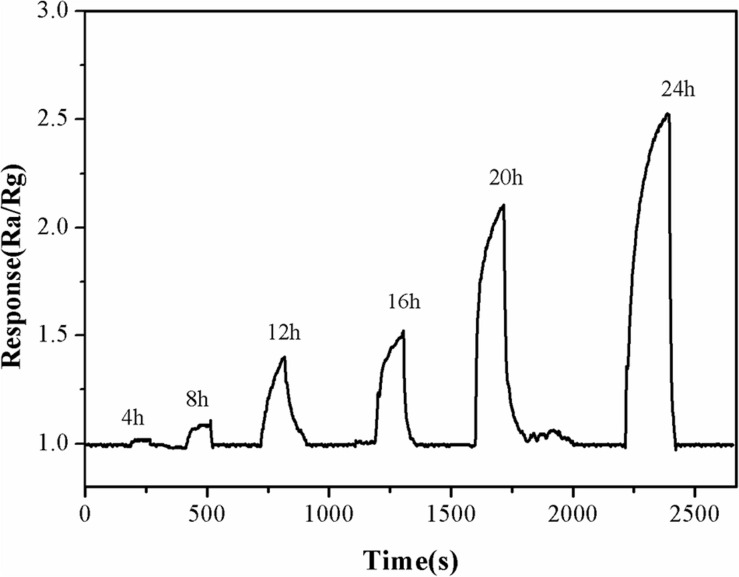
The response and recovery curves of α-Fe_2_O_3_ sensor to TMA gas released from dead fish at 250°C for 24 h.

Fish freshness was detected using α-Fe_2_O_3_ sensor to investigate the practical application of α-Fe_2_O_3_ sensor. As can be seen from Figure 11, almost no TMA was released within 4 h after the carps died. TMA began to be released at 8 h. Subsequently, as the time extended, an increasing concentration of TMA was observed. The freshness of the fish also deteriorated, and they began to rot by 12 h., as detected by their characteristic odor. The fish’s eyes and the color of the fish become dark By 24 h. These changes in color and odor confirmed that the fish were inedible. At this point, buyers can make accurate judgments based on sight and smell; therefore, the detection was terminated at 24 h. Based on these observations, we confirmed that the developed α-Fe_2_O_3_ sensor could be used as a gas-sensitive material to test the freshness of fish by detecting TMA gas in a non-destructive manner.

## Conclusion

Herein, the application of Fe_2_O_3_ nanospheres as a gas sensor to detect fish freshness was explored. Our results confirmed that the α-Fe_2_O_3_ sensor responded to TMA gas within 1 min, with a recovery time less than 4 min. Moreover, the sensor showed a good linear relationship (*R* = 0.9974) between the response value to TMA and its concentration. Importantly, the α-Fe_2_O_3_ sensor could be used to effectively detect fish freshness as a gas sensor. The present study made several notable contributions to application of α-Fe_2_O_3_ sensor to the field of environmental protection. Contamination causes serious pollution of fish and other aquatic products, which are harmful to human health if consumed. As a gas sensor, the Fe_2_O_3_ nanospheres could also be used to monitor environmental pollutants. A limitation of this study is that we only tested one types of fish, and the research was not complete, for example, the developed sensor requires further refinement such that it could be used in the market or supermarket to assess fish freshness. Further work needs to be done to establish more convenient and sensitive detection methods to reduce the harm to human health caused by environmental contamination of fresh fish.

## Data Availability Statement

All datasets generated for this study are included in the article/supplementary material.

## Ethics Statement

The animal study was reviewed and approved by Animal Ethical Care Committee of Qiqihar Medical University. Written informed consent was obtained from the owners for the participation of their animals in this study.

## Author Contributions

All the authors participated in this work. WQZ conceived the study. LL and SF analyzed the data and wrote the manuscript. LY, LF, HY, and XLi helped with experiments and provided suggestions in writing. XG, WBZ, WZ, and XLv designed and performed the experiments.

## Conflict of Interest

The authors declare that the research was conducted in the absence of any commercial or financial relationships that could be construed as a potential conflict of interest.
